# Effects of 660 nm & 808 nm photo-biomodulation on S100A8/A9 in symptomatic pulpitis: a randomized controlled trial

**DOI:** 10.1007/s10103-026-05006-z

**Published:** 2026-08-29

**Authors:** Meifang Zhu, Eliza Ranjit, Carlos Marcelo Silva Figueredo, Laurence James Walsh, Roy George

**Affiliations:** 1 Griffith University, Gold Coast, Australia; 2 The University of Queensland, UQ Oral Health Centre, Herston, Australia

**Keywords:** Photo-Biomodulation, Low Level Light Therapy, Lasers, Dental Pulp, Biomarkers, S100 A8/A9

## Abstract

Photobiomodulation (PBM) modulates inflammation and promotes tissue repair. S100A8/A9 is a neutrophil-derived calcium-binding heterodimer that acts as a pro‑inflammatory damage‑associated molecular pattern, serving as a biomarker of inflammation. This study aimed to evaluate the effects of PBM at 660 nm and 808 nm wavelengths on S100A8/A9 levels in pulp blood (PB) and gingival crevicular fluid (GCF) in patients with symptomatic irreversible pulpitis (SIP). Patients diagnosed with SIP were block-randomised into three groups: Group 1 received 660 nm PBM (*n* = 14), Group 2 received 808 nm PBM (*n *= 13), and Group 3 received sham irradiation (*n* = 8). PB and GCF samples were collected pre- and post-irradiation. Laser activation lasted 60 s, delivering energy densities of 17.26 J/cm² (660 nm) and 33.16 J/cm² (808 nm). S100A8/A9 levels were quantified via enzyme-linked immunosorbent assay (ELISA) and analysed using fold change from baseline, Spearman non-linear correlation analysis for age effects, Fisher’s exact test, and Kruskal-Wallis tests (α = 0.05). There was inter‑individual variation in baseline S100A8/A9 levels in both PB and GCF. Baseline S100A8/A9 levels were higher in GCF than in PB at the individual level, with no age effect in PB, whereas GCF levels were lower in the 60–80‑year group than in the 45–59‑year group. PBM at 660 nm and 808 nm produced numerically greater fold increases in S100A8/A9 in PB and GCF than sham, particularly for 660 nm in older participants, but these effects did not reach the threshold for statistical significance. This study is the first clinical trial to explore the effect of S100A8/A9 expression in PB and GCF following a single PBM treatment at wavelengths of 660 nm and 808 nm. This pilot study demonstrated that S100A8/A9 can be detected in pulp blood and GCF from patients with SIP and may serve as a potential biomarker for PBM response, although no statistically significant differences were found. Given the cumulative and time-dependent nature of PBM effects, future research should investigate multi-dose protocols to better define its role in managing pulpal inflammation.

**Supplementary Information:**

The online version contains supplementary material available at https://doi.org/10.1007/s10103-026-05006-z.

## Introduction

Dental pulp serves multiple biological roles, including nutritional support for odontoblasts, sensory functions, immune defense against noxious stimuli, and stimulation of tertiary dentin formation [1]. When exposed to irritants caused by caries, trauma, or fractures, the pulp triggers a cascade of inflammatory processes [2]. Neuropeptides and inflammatory mediators are released to help tissue repair [3], severe inflammation eventually cause irreversible pulp damage, clinically known as symptomatic irreversible pulpitis [4].

PBM enhances cellular function and tissue healing by modulating inflammation, reducing inflammatory mediators, improving blood flow, accelerating regeneration and providing analgesia [5–7]. PBM is recognized as an adjunct or standalone treatment for minimally invasive treatments of pulp inflammation [8].

Current clinical research on the anti-inflammatory effects of PBM as an adjunct to endodontic treatment for SIP has primarily focused on intraoperative and postoperative pain severity [9]. While most studies report consistent pain reduction from PBM, patient-reported outcomes are inherently subjective and lack objective, quantitative assessment [10]. To address this, inflammatory biomarkers have been proposed to evaluate PBM effects on pulp tissue, though few studies have pursued this. Montoro et al. (2014) demonstrated in vitro that PBM (855 nm, 15 J/cm²) reduced nitric oxide and reactive oxygen species in lipopolysaccharide treated pulp cells [11]. In vivo, Silva et al. (2022) found that PBM (808 nm, 3 J) decreased interleukin-23 levels in rat pulp exposed to bleaching-induced stress, but hypoxia-inducible factor (HIF)-1α expression remained unchanged [8]. These findings highlight the potential of biomarker-based approaches to objectively assess PBM-mediated modulation of pulpal inflammation.

S100 A8/A9 are calcium-binding molecules that play crucial roles in innate and acquired immune defense processes [12, 13], including cell cycle progression and differentiation, calcium homeostasis, and cytoskeleton metabolism [14]. S100 A8/A9 is expressed mostly by infiltrating neutrophils and phagocytes [15] and associated with tissue damage and inflammation [16, 17]. S100 A8/A9 has multifaceted roles in modulating inflammation, with both pro- and anti-inflammatory dualistic biological functions [17]. S100 A8/A9 are widely recognized for its involvement in inflammation and used as a biomarker, yet only few laboratory studies have specifically evaluated its utility as a biomarker in pulp diseases [18, 19]. Past work has relied on complex gene profiling and immunohistochemical analyses [18, 19], which may limit its clinical applications as possible biomarker.

This study measured S100 A8/A9 levels as a means of exploration of the effects of PBM on the pulp tissue, by tracking the levels of S 100A8/A9 before and after PBM in two wavelength 660 nm and 808 nm, in PB and in GCF, in patients with SIP. The null hypothesis is that there is no difference in S100A8/A9 expression following PBM in PB and GCF.

## Methodology

### Ethical approval

Griffith University’s human ethics committee approved this study (Ethical approval number 2022/670). The study was registered with the Australian and New Zealand Clinical Trials Registry (ACTRN12625000022460).

### Sample size

A pilot study was conducted to gather preliminary data and to validate the study protocol. The mean and standard deviation from the pilot study were used to calculate effective sample size as 7 subjects in each group.

### Participant selection

Patients diagnosed with SIP at Griffith University Dental Clinic were invited. The study protocols were explained and written consent was obtained.

### Inclusive criteria

Patients aged 18–80 who diagnosed with SIP were included. Diagnosis followed the American Association of Endodontists guidelines and Wolters et al. (2017). Patients reporting a pain score of 7/10 or higher using visual analogue scale, experiencing lingering pain to thermal stimuli, sharp to dull throbbing sensations, and sleep disturbances were diagnosed as SIP [20].

### Exclusion criteria

Periodontal disease, cancer, immunocompromised condition, taking opioid medications, root resorption, history of dental trauma or root fracture, open apices or apical radiolucency cases were excluded.

### Randomisation and study design

Patients were randomized in blocks of three into three study groups. Group 1 received PBM at 660 nm (*n* = 14), Group 2 received PBM at 808 nm (*n* = 13), and Group 3 received sham irradiation as the control group (*n* = 8). Pulp blood (PB) and gingival crevicular fluid (GCF) samples were collected before and after the intervention using a standardized protocol.

A total of 51 participants were enrolled and randomized. However, 15 participants were excluded from the final analysis because adequate pulp blood samples could not be obtained due to calcific changes within the pulp chamber and/or fibrosis of the pulp tissue. These post-randomization exclusions resulted in unequal group sizes.

Owing to the nature of the intervention, the researcher administering PBM could not be blinded. However, participants and the investigator performing the ELISA analyses remained blinded to group allocation. The study was conducted and reported in accordance with the CONSORT guidelines (Supplementary S1).

### PB and GCF collection

Patients were treated by one postgraduate student undertaking endodontic specialty study. Patients received local anesthesia by infiltration or nerve block using 2% lidocaine hydrochloride with 1: 80,000 epinephrine (Septodont, Saint-Maur-des-Fosses, France).

Preoperative GCF samples were collected using GCF collection strips (PerioPaper®, Oralflow, NY, US), by positioning the strip into the gingival sulcus of the tooth for 30 s. This sample was taken before the PBM intervention (GCF-Before). The strips were placed into an Eppendorf tube with 1 ml of 1X phosphate buffer solution (PBS) solution (Sigma Aldrich, St Louis, MO, USA).

Teeth distal and mesial to the tooth receiving treatment were isolated with rubber dam. The rubber dam and teeth were swabbed using sterile gauze soaked with 1% sodium hypochlorite solution (Endosure™, Dentalife, Australia) for 30 s and then rinsed with sterile water. Under an operating microscope (Global Surgical Corporation, USA), sterile diamond burs were used to remove restorations and caries under water cooling. A new sterile diamond bur was then used to access the pulp chamber. Following access, a sterile paper point (WaveOne Gold Absorbent Points, medium, Dentsply Maillefer, Switzerland) was dipped into the pulp chamber and left in place until a 5 mm length of the paper point was saturated with pulpal blood. This blood-saturated paper point collected before the PBM intervention sample (PB-Before) was placed into an Eppendorf tube with 1 ml of 1X PBS solution.

Following the PBM treatment and 60 s waiting period, a fresh sterile paper point was inserted into the pulp chamber to collect the post-intervention sample (PB-After) and a further PerioPaper® strip was used to collect a GCF sample (GCF-After). Subsequently, pulp extirpation was performed.

### Sample storage

After PB and GCF samples were collected, Eppendorf tubes were kept at room temperature for 60 min, then vortex mixed, paper points and PerioPaper® strips were removed using sterile tweezers. Eppendorf tubes were centrifuged at 5000 rpm for 5 min, the supernatant stored frozen at -20℃.

### PBM protocol

PBM was performed by placing the optical fibre tip in line with the occlusal access cavity of the tooth with exposed pulp tissue. Details of the laser light sources are given in Table 1. The exposure time was 60 s. Gingival tissues were not in the direct line of irradiation. The power output of two lasers was determined using a laser power meter (Ophir Optronics, MKS Instruments Inc, Utah, USA). PBM at 808 nm was administered with a power density of 0.55 W/cm² and an energy density of 33.16 J/cm², while PBM at 660 nm was administered with a power density of 0.29 W/cm² and an energy density of 17.26 J/cm².

**Table 1:** PBM Parameters

Parameters	Value	Value
Type of laser	808 nm Diode	660 nm Diode
Laser Manufacturer	Konftec™ Klas-DX KLAS-DX82	Konftec™ Klas-DX KLAS-DX61
Mode of operation	Continuous wave	Continuous wave
Method of application	Non-contact	Non-contact
Delivery system	Optical fiber	Optical fiber
Displayed Power (Measured Power)	250 mW (210 mW)	150 mW (110 mW)
Measured energy dose for 60 s	12.6 J	6.56 J
Power density	0.55 W/cm^2^	0.29 W/cm^2^
Spot size in mm	Optical Cap-E laser probe 7 mm	Optical Cap-E laser probe 7 mm
Spot area in cm^2^	0.38 cm^2^	0.38 cm^2^
Duration	60 s	60 s
Energy density	33.16 J/cm^2^	17.26 J/cm^2^

### ELISA

The S100 A8/A9 protein level was determined using a commercial ELISA kit (R&D Human S100A8/S100A9 Heterodimer DuoSet ELISA assay kit, DY8226-05, Biotechne, USA). All assays were performed in duplicate. Samples were thawed to room temperature (RT, 22 °C) and were diluted 500 times in the supplied diluent. The optimal dilution of 1/500 was based on pilot testing of a dilution series (1/100, 1/500, 1/1000, 1/2000). The assay plates were coated with 100 µL of capture antibody. The plates were sealed and incubated overnight at RT. The plates were washed three times with the wash buffer, then 300 µL/well of Block Buffer was added, followed by incubation at RT for 60 min and three cycles of washing. Then, 100 µL of diluted samples (1/500) was added into the wells, in duplicates, and the plates were incubated at RT for 120 min. After 3 cycles of washing, 100 µL of the detection antibody was added, and the plates were incubated for 120 min. After washing, 100 µL of Streptavidin-horseradish peroxidase was added, and the plates were incubated for 20 min at RT in the dark. After washing, 100 µL of the supplied substrate solution was added, and the plates were incubated at RT for 20 min in the dark. After this time, 50 µL of the supplied stop solution was added and mixed by gently tapping the plate. Absorbance readings were then obtained using a microplate reader (Multiskan SkyHigh, ThermoFisher Scientific, USA) at a wavelength of 450 nm.

S100 A8/A9 levels were calculated by interpolation from the standard curve, corrected for the dilution factor of 500, and expressed in ng. Actual levels before and after PBM, and fold changes from baseline were also calculated. Data for S100 A8/A9 levels were presented before and after the PBM interventions as box and whisker plots.

### Statistical analysis

Statistical analyses were performed using GraphPad Prism version 10.4.2 (GraphPad Software, San Diego, CA, USA) and Microsoft Excel. Demographic variables (age, gender, tooth type and cause of SIP) were assessed using non-parametric analysis of variance.

Given the observed variation in age distribution across the intervention groups, age was included as a covariate in the statistical analysis. Fold-change values (post-intervention/pre-intervention) were calculated for S100A8/A9 levels in both PB and GCF samples and used as the primary outcome measure. Analysis of covariance (ANCOVA) was performed with fold change as the dependent variable, intervention group (660 nm PBM, 808 nm PBM, and sham control), and age as a continuous covariate.

Associations between baseline S100A8/A9 levels in pulpal blood (PB) and gingival crevicular fluid (GCF), as well as their relationship with age, were examined using Spearman’s rank correlation analysis. Fold-change data were visualised using box-and-whisker plots. Differences in median percentage change in S100A8/A9 across groups were assessed using the Kruskal–Wallis test. Fisher’s exact test was used to compare the proportion of samples demonstrating an increase in S100A8/A9 levels between intervention groups. The threshold for statistical significance was set at *p* < 0.05.

## Results

A total of 36 participants were included in the final analysis across the three intervention groups, with ages ranging from 23 to 78 years. Fifteen participants were excluded after randomization because adequate pulp blood samples could not be obtained. The demographic characteristics are presented in Table 2. A statistically significant difference in age distribution was observed between groups (*p* = 0.023), while no significant differences were detected for gender, tooth type, or cause of symptomatic irreversible pulpitis.

**Table 2:** Demographic characteristics of subjects diagnosed with SIP. Baseline demographic and clinical characteristics of patients in the 660 nm PBM (*n* = 14), 808 nm PBM (*n* = 13), and sham (*n* = 8) groups. Values are mean ± SD or n (%); *p*-values from one-way ANOVA (age) and chi-square/Fisher's exact test (categorical variables)

		660 nm (*n* = 14)	808 nm (*n* = 13)	Sham control (*n* = 8)	*p*-value
Age, mean ± SD		55.2 ± 15.2	44.2 ± 17.1	36.5 ± 10.9	0.024*
Gender, *n* (%)	Male	5 (35.7%)	6 (46.2%)	2 (25%)	0.615
	Female	9 (64.3%)	7 (53.8%)	6 (75%)	
Tooth type, *n* (%)	Anterior	1 (7.14%)	1 (7.7%)	0 (0%)	0.717
	Premolar	2 (14.3%)	1 (7.7%)	0 (0%)	
	Molar	11 (78.56%)	11 (84.6%)	8 (100%)	
	Deep caries lesion	7 (50%)	8 (61.5%)	5 (62.5%)	0.783
Causes of SIP, *n* (%)	Leakage of deep filing	3 (21.4%)	2 (15.4%)	2 (25%)	
	Crack	3 (21.4%)	2 (15.4%)	1 (12.5%)	
	Trauma	0	1 (7.7%)	0	
	Traumatic occlusion	1 (7.1%)	0	0	

* = *p* < 0.05

One participant from the 808 nm PBM group was excluded because the S100A8/A9 concentration exceeded the assay detection range.

**Fig. 1:**
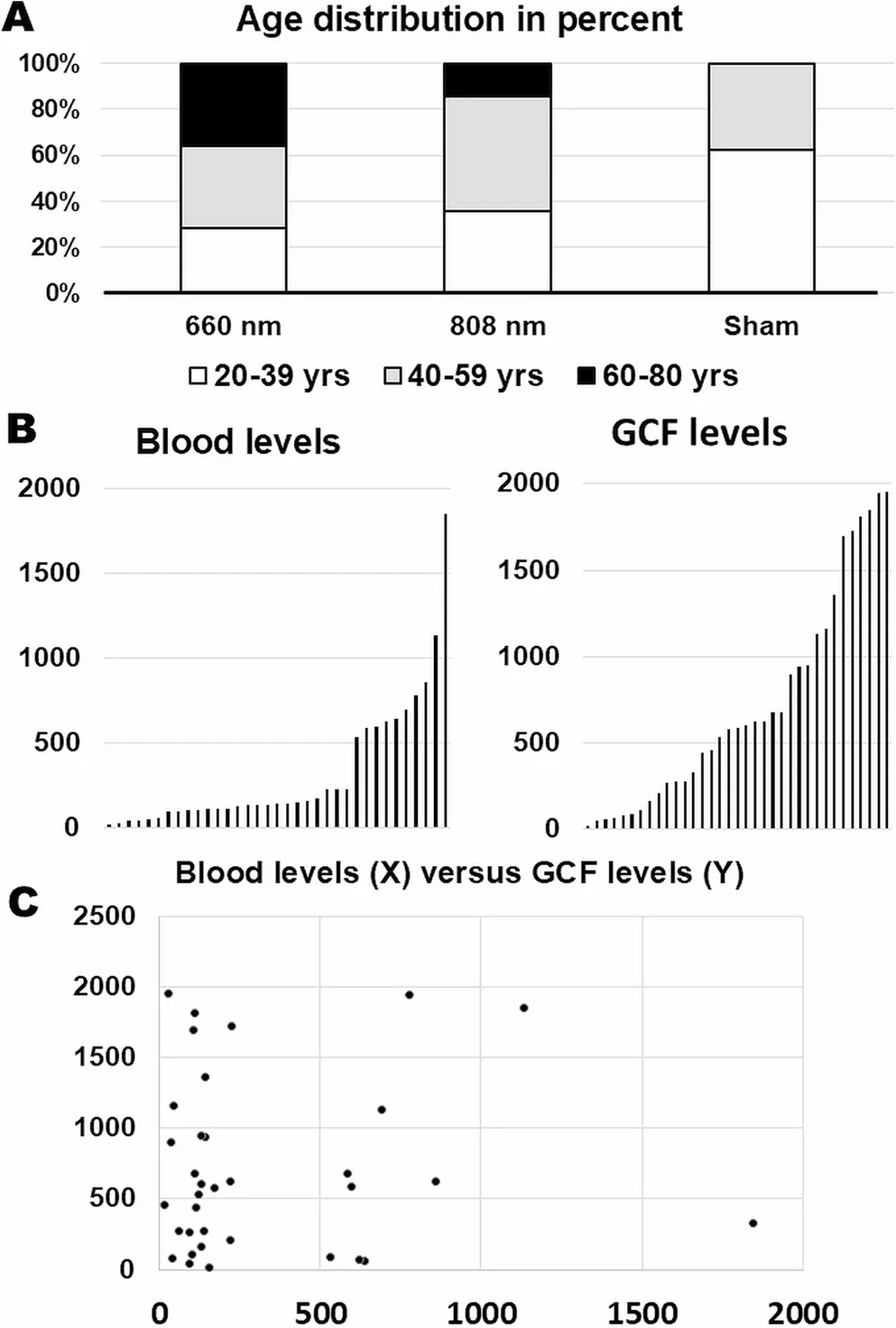
Baseline participant characteristics and S100A8/A9 levels prior to intervention. (**A**) Age distribution across the 660 nm PBM, 808 nm PBM, and sham control groups. (**B**) Baseline S100A8/A9 concentrations in peripheral blood (PB) and gingival crevicular fluid (GCF), demonstrating inter-individual variability and generally higher levels in GCF. (**C**) Relationship between baseline PB and GCF S100A8/A9 concentrations. PB, peripheral blood; GCF, gingival crevicular fluid; PBM, photobiomodulation

Baseline participant characteristics and S100A8/A9 levels are presented in Fig. 1. The age distribution differed between treatment groups (Fig. 1A). Baseline S100A8/A9 concentrations demonstrated substantial inter-individual variability in both PB and GCF samples (Fig. 1B). No correlation was observed between baseline PB and GCF S100A8/A9 concentrations at the individual level (Fig. 1C).

**Fig. 2:**
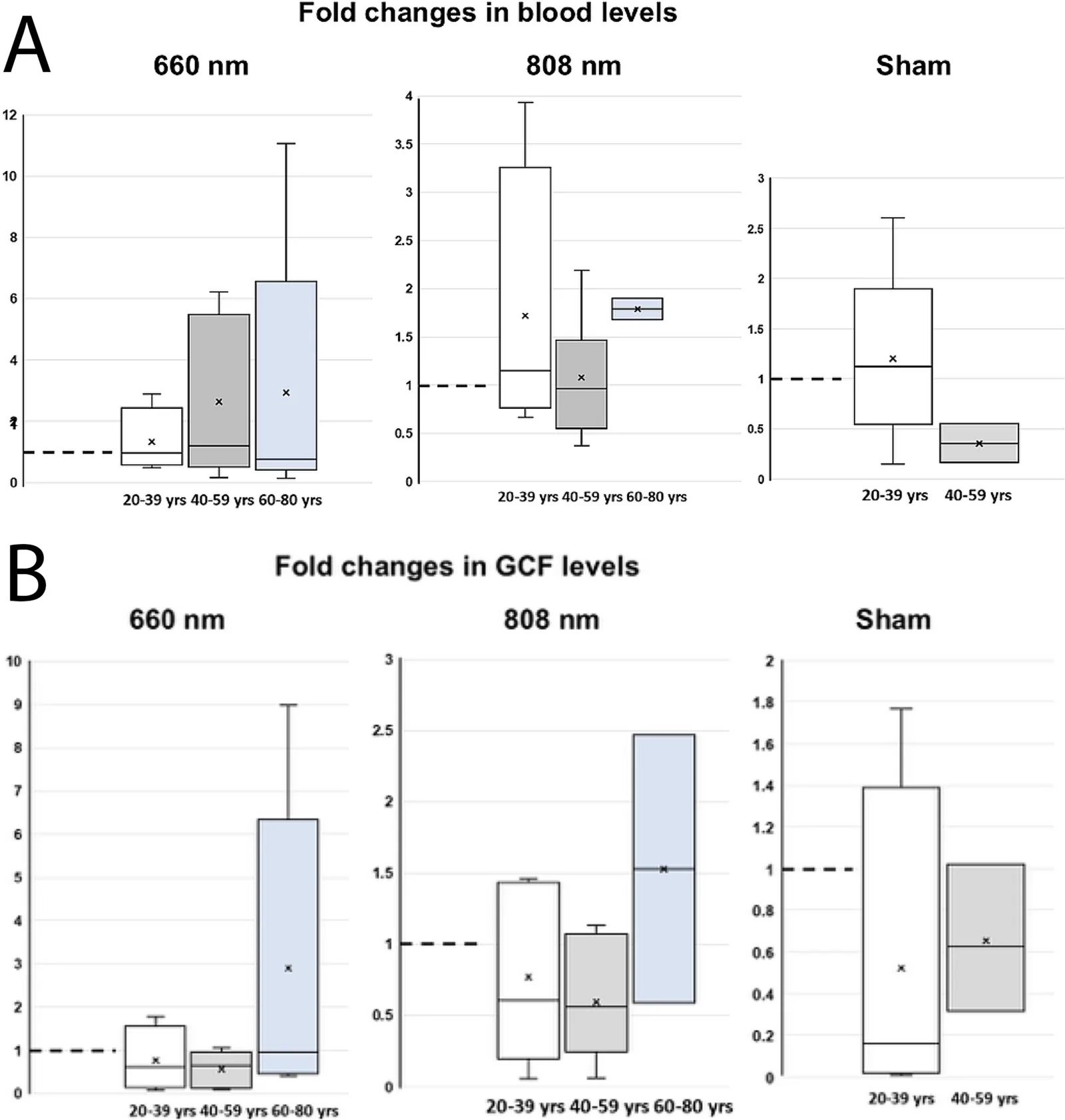
Fold-change in S100A8/A9 levels following intervention by age group and treatment (660 nm PBM, 808 nm PBM, and sham control). (**A**) Peripheral blood (PB) and (**B**) gingival crevicular fluid (GCF) S100A8/A9 fold-change values (post-intervention/pre-intervention). Data are presented as box-and-whisker plots, where × indicates the mean, boxes represent the interquartile range, and whiskers represent the 95% confidence intervals. The dashed horizontal line indicates a fold change of 1 (no change from baseline). Note that each panel is displayed using an independent y-axis scale

Changes in S100A8/A9 levels following PBM were evaluated using fold-change values (Fig. 2). Fold-change distributions in both PB and GCF samples showed considerable inter-individual variability and substantial overlap between treatment groups. To account for the observed differences in age distribution between groups, ANCOVA was performed with age included as a continuous covariate. In PB samples, ANCOVA demonstrated no significant effect of intervention group (F = 0.68, *p* = 0.51) and no significant effect of age (F = 1.99, *p* = 0.17). Similarly, in GCF samples, no significant effect of intervention group (F = 0.49, *p* = 0.62) or age (F = 1.40, *p *= 0.25) was observed.

Fisher's exact test showed no significant differences in the proportion of samples demonstrating increased S100A8/A9 levels between the 660 nm PBM, 808 nm PBM, and sham control groups (Table 3; all* p* > 0.05). Similarly, the Kruskal–Wallis test revealed no significant differences in median percentage change in S100A8/A9 levels among the three intervention groups (Table 4), and the null hypothesis was therefore retained.

**Table 3:** Comparison of the ratio of patients showing an increase versus decrease in S100A8/A9 levels in PB and GCF, by groups. *P*-values were calculated using Fisher’s exact test

Group	*n*	Increase/ Decrease ratios	*P* value
PB 660 nm vs. control	14/8	2.57	0.3999
PB 808 nm vs. control	13/8	3.00	0.1827
GCF 808 nm vs. control	13/8	1.04	1.0000
GCF 660 nm vs. control	14/8	0.710	1.000
PB 660 nm vs. PB 808 nm	14/13	0.535	0.6951
GCF 660 nm vs. GCF 808 nm	14/13	0.704	1.0000

**Table 4:** Comparison of percentage changes in S100A8/A9 levels in PB and GCF among the 660 nm PBM, 808 nm PBM, and sham groups. Data are presented as median (minimum, maximum) percentage change and mean rank. Kruskal–Wallis test was used for between-group comparisons

Percentage differences	660 nm Group Rank *n* Median% (Min, Max)	808 nm Group Rank *n* Median% (Min, Max)	Sham Group Rank *n* Median% (Min, Max)	χ2	*p*
PB	*n* = 14 -24.34 (-99.51, 1266.07) 17.08	*n* = 13 13.32 (-66.54, 327.57) 19.08	*n* = 8 -32.91 (-68.17, 140.90) 13.75	1.462	0.482
GCF	*n* = 14 -57.06 (-92.49, 23.21) 13.75	*n* = 13 -68.87 (-99.07, 42.24) 12.60	*n* = 8 -68.70 (-99.27, 76.41) 12.71	0.123	0.940

## Discussion

This is the first clinical study to report S100 A8/A9 levels in PB and GCF for teeth with SIP receiving a single dose of PBM. The S100 A8 and A9 proteins have been studied in the context of systemic and pulp inflammatory conditions [21, 22]. Previous studies of S100 A8/A9 in pulp tissue were done by gene profiling and immunochemical staining [18, 19]. Jungbluth et al. (2022) reported an increased expression of S100 A8 in SIP compared to normal and asymptomatic pulp tissue [18]. The same group reported a significant increase in S100 A8 and A9 levels in the pulp stroma in SIP, implying their potential use as biomarkers for diagnosing pulp diseases or for monitoring therapy outcomes [19].

Compared to Jungbluth’s sampling method of obtaining the entire pulp tissue, the present study collected pulp blood samples using paper points. The present investigation also included a GCF sample, which could be a non-invasive and cost-effective method for assessing the level of mediators [23, 24].

Previous studies have shown high variability pulpal blood S100A9 levels ranging from 150 to 180 ng/mL [25], 500–1500 ng/mL [26], and 50–3540 ng/mL [27]. In line with previous reports, the present study also observed considerable inter‑individual variability in S100A8/A9 levels. Moreover, the lack of correlation between PB and GCF S100A8/A9 suggests that expression varies between tissues. Although ageing alters pulp anatomy and vascularity through increased calcification, no association was observed in this study between age and baseline PB S100A8/A9 levels. In GCF, baseline S100A8/A9 showed an age-related pattern, with lower levels in the 60–80-year group than in 45–59-year-olds, which may reflect age-related periodontal tissue changes. However, given the small sample size, this age effect should be interpreted cautiously, and needs to be validated in larger cohort studies.

When comparing baseline S100 A8/A9 levels in PB and GCF samples, there were higher S100 A8/A9 levels in GCF than in PB. The higher level of S100 A8/A9 in GCF samples versus pulp blood can be attributed to the role of GCF as an inflammatory exudate.

Fold-change analysis (post- vs. pre-intervention at the individual level) suggested effects of both age and treatment on S100A8/A9 levels. Across age groups, 660 nm PBM produced the largest increases in both PB and GCF compared with 808 nm and sham, with the greatest responses in aged ≥ 60 years. Although these age- and treatment-related differences in fold change did not reach statistical significance, the current results inspire future research in larger sample group.

PBM has been reported to be effective in activating neutrophils at inflammation sites, releasing S100 A8/A9 from their cytoplasm [28]. This process is linked to PBM’s ability to modulate cellular activity through its effects on mitochondria and reactive oxygen species (ROS) production [6]. When applied to inflamed pulp tissues, PBM can stimulate neutrophils, which contain large amounts S100 A8/A9 [29]. Activation of neutrophils by PBM can trigger the release of S100 A8/A9 through alternative secretion pathways, such as the formation of neutrophil extracellular traps (NETs) [30]. This release occurs rapidly, which, in the current study, possible explains the increase in levels in PB was detected immediately after a single dose of PBM. The increase may reflect the mobilisation of existing cellular stores, rather than changes in gene expression or protein synthesis. The released S100 A8/A9 may then act as a damage-associated molecular pattern [31], further amplifying the inflammatory response and neutrophil activation in a dose-dependent manner [30]. This pathway may explain how PBM could be a therapeutic approach for modulating local immune responses in SIP and other inflammatory conditions.

S100 A8/A9 modulates the production of pro-inflammatory mediators such as cytokines, chemokines, ROS, and nitric oxide (NO), thereby reducing inflammation and oxidative damage [17]. Thus, numerical level changes of S100 A8/A9 observed after PBM in this study may indicate PBM initially upregulate host responses in the inflamed pulpal tissue as a prerequisite for later tissue healing, repair, or regeneration [32]. Therefore, the absence of statistically significant findings in this study may suggest as an indication that any potential effect may be modest and relative to inter-individual variability under a single-dose protocol. Hence subsequent robust trials should explore multi-dose or varied-frequency delivery protocols.

The biological effect of PBM in various energy dosages on dental tissue was reported previously [9]. Sleep et al. (2022) examined the bioenergetic effects of photobiomodulation on osteoblast mitochondria derived from human dental pulp stem cells [33] and found that optimal bio stimulation results were observed at lower energy densities (0.5–4 J/cm²), while higher densities (16 J/cm²) showed inhibitory effects [33]. However, the impact of energy density could vary by cell type. A further factor is heterogeneity in laser parameter reporting, which can introduce bias [33]. In the present study, single dose PBM at wavelengths of 660 nm and 808 nm, using energy densities of 16.8 J/cm² and 31.73 J/cm², respectively, fall within the previously reported effective range [33, 34]. It is important to note that repeated PBM doses may yield larger and more sustained cellular effects [35]. However, the nature of SIP treatment in this study made the administration of multiple doses impractical. Additionally, studies using multiple near-infrared light sources at wavelengths from 700 to 1100 nm have also reported biological effects that are both dose- and time- dependent [35]. In the present study, the different changes observed in the 660 nm group may also related to the shallower penetration depth of 660 nm compared with 808 nm, due to sampling from superficial pulpal tissue. Future studies should therefore investigate the effects of multi-dose protocols and different wavelength PBM regimens in the management of SIP.

In the present study, GCF samples did not demonstrate comparable responses to PBM as PB did when the pulp tissue was the treatment target. Consequently, using GCF samples to assess inflammatory biomarkers as a reflection of pulp tissue status may be questionable, especially when attempting to record the immediate effects of PBM treatment when the GCF sampling site is not in the line of fire for the laser.

The present study has several limitations. The overall sample size was small, and the control group contained fewer participants overall and no subjects in the oldest age group (60–80 years) than the two PBM intervention groups. This inequality limits the robustness of between‑group comparisons. Future studies with larger samples with more balanced group sizes and age distributions is recommended. Methodologically, collection of PB with paper points is simple and cost‑effective, but may introduce variability in sample volume and thus in biomarker levels. Moreover, administration of local anaesthetic solutions whether or not they contain epinephrine prior to sampling may alter pulpal and GCF blood flow and consequently S100A8/A9 levels. Future research should evaluate the effects of sampling technique and local anaesthetic use when assessing S100 A8/A9 levels in PB and GCF.

## Conclusion

This clinical pilot study evaluated the effects of single-dose PBM at 660 nm and 808 nm wavelengths on S100A8/A9 levels in PB and GCF in patients with SIP. Baseline S100A8/A9 levels were assessed across tissue types, age groups, and individual variability, with post-to-pre-intervention fold changes subsequently analyzed.

The observed trends, with the lack of statistically significant differences between groups, may be influenced by the small and imbalanced sample sizes and the use of a single-dose PBM protocol. Nevertheless, these preliminary findings provide valuable methodological insights for future research. Further studies with larger, age-matched cohorts and optimised PBM dosing protocols are required to better define the therapeutic potential of PBM in modulating pulpal inflammation.

## Supplementary Information


Supplementary Material 1.


## Data Availability

No datasets were generated or analysed during the current study.
