# Supplementary material for: Effects of 660 nm & 808 nm photo-biomodulation on S100A8/A9 in symptomatic pulpitis: a randomized controlled trial
Source: Lasers Med Sci. 2026 Aug 29;41(1):206. doi: 10.1007/s10103-026-05006-z (PMC13526045; doi:10.1007/s10103-026-05006-z)
Supplement: Supplementary file 1 [file 10103_2026_5006_MOESM1_ESM.docx]

**CONSORT DIAGRAM**

Assessed for eligibility and enrolled (n=51)

Randomised (n=51)

Allocation (n=36)

660nm(n=14)

808nm(n=14)

sham (n=8)

Elisa analysis:144 sample from 36 subjects

Statistical anaylse (n=35)

660nm (n=14)

808nm(n=13)

shame(n=8)

PBM: Photobiomodulation; nm: nanometres; ELISA: Enzyme-Linked Immunosorbent Assay.

Excluded after ELISA (n = 1)

Reason: S100A8/A9 level exceeded assay detection range (in 808 nm group)

Excluded after randomisation (n = 15)

Reason: insufficient pulp blood collection due to fibrotic/calcific canal change
